# Effect of a stylet on specimen sampling in thyroid fine needle aspiration: A randomized, controlled, non-inferiority trial

**DOI:** 10.3389/fendo.2023.1062902

**Published:** 2023-03-23

**Authors:** Pengfei Luo, Xiali Mu, Wei Ma, Dahai Jiao, Peixin Zhang

**Affiliations:** Department of General Surgery, Fuyang People’s Hospital, Fuyang, China

**Keywords:** fine needle aspiration, thyroid nodule, specimen adequacy, stylet, needle

## Abstract

**Background:**

There is a cost advantage in using a needle without stylet over a needle with stylet in thyroid fine needle aspiration (FNA). This study aimed to elucidate the non-inferiority of thyroid FNA without a stylet (S-) to thyroid FNA with a stylet (S+) on specimen sampling.

**Methods:**

In this study, patients with thyroid nodules undergoing FNA were consecutively enrolled between May 2022 and July 2022. One experienced operator performed two punctures of each nodule with a stylet and without a stylet. Specimen adequacy was the primary outcome. Wald test was used for statistical analysis of the primary outcome. The difference in specimen adequacy between the two methods was expressed as a two-sided 95% confidence interval (CI). The S- method was considered non-inferior to the S+ method if the lower bound of the 95% CI of the S- minus S+ adequacy difference was greater than a predetermined non-inferiority margin of -10%.

**Results:**

A total of 149 patients (195 nodules) were enrolled in the study. A total of 167 of 195 nodules (85.64%) and 169 of 195 nodules (86.67%) were obtained adequate specimens using the S+ and S- methods, respectively. The difference in specimen adequacy (S- minus S+) between the two methods was 1.03% (95% CI, -5.83% to 7.88%). The lower bound 95% CI of the difference in specimen adequacy (-5.83%) was greater than the predetermined non-inferiority margin of -10%. The difference in the yield for malignancy was not significantly different between the two methods.

**Conclusion:**

Thyroid FNA without a stylet is non-inferior to thyroid FNA with a stylet on specimen sampling.

## Introduction

Fine needle aspiration (FNA) is a simple, minimally invasive, and highly accurate method widely used in the diagnosis of many organ diseases, including thyroid (1–13). A stylet can theoretically prevent the lumen of the needle from being blocked by other non-lesion components, including blood, before puncturing into the target lesion, thus allowing fuller aspiration of the target tissue into the needle once the stylet is removed. The stylet is routinely used by many puncturing physicians to improve the quality of specimens (1, 2). Although this assumption of a preference for the use of a stylet seems logical, this assumption has not been demonstrated on an empirical basis. Several studies in other areas of FNA, such as gastrointestinal endoscopy and respiratory endoscopy FNA have shown that the stylet does not improve specimen quality and diagnostic efficiency (3–8). Besides, some studies have shown that stylet may be associated with an inferior specimen quality (9).

Furthermore, the value of the stylet has not been systematically evaluated in the FNA of the thyroid. Only one study evaluated the role of stylet using a few selected nodules (hypoechoic vascular type II nodules) (1), representing a small fraction of all types of thyroid nodules (14). This study aimed to compare the specimens obtained by FNA with and without the stylet in all types of unselected thyroid nodules for adequacy and yield for malignancy.

## Materials and methods

### Study design

A single-center, prospective, randomized, controlled, non-inferiority trial was conducted at Fuyang People’s Hospital, a tertiary referral medical center. Written informed consent was obtained from all patients, and the study was approved by the Ethics Committee of Fuyang People’s Hospital (NO: 2022-80).

### Patients

Patients with thyroid nodules who underwent FNA between May 2022 and July 2022 were prospectively enrolled in the study. The inclusion criteria were: (1) patients aged 18 years and above; (2) patients who provided written informed consent; and (3) patients who underwent ultrasound suggesting the presence of a thyroid nodule. The exclusion criteria were: (1) patients under anticoagulant therapy, such as aspirin and warfarin; (2) patients who could not cooperate with punctures, such as severe cough; and (3) those who could not provide written informed consent.

### Randomization

Each nodule was sampled for four passes (twice with a stylet (S+) and twice without a stylet (S-)). The nodules were randomly divided (1:1) into S+ (order of the four passes; S+→S-→S+→S-) and S- (order of the passes; S-→S+→S-→S+) to avoid the effect of bleeding from the previous pass on the later pass specimen. The order of S+ or S- first pass was determined by a preprinted random sequence that was kept in an opaque sealed envelope that the operator opened after the patients confirmed their enrollment.

### FNA procedure

FNAs were performed under ultrasound (US) guidance by the same experienced operator. For patients with multiple nodules, FNA was performed on suspicious nodules, otherwise, the largest nodule was selected for sampling. For patients with mixed cystic-solid nodules, FNA was performed from the solid component. An US scanner (M9, Mindray, Shenzhen, China) was used for US with a 7.5-15 MHz linear array transducer. The medical record report contained the number, location, size, echogenicity, composition, vascularity, calcifications, depth, and Thyroid Imaging Reporting and Data System (TIRADS) categories of the punctured nodules. The passes were performed using a 25-gauge disposable puncture needle with a stylet (CCZA, Leapmed, Suzhou, China) without syringes. The stylet in the needle was kept during the puncture for S+ passes and removed before S- passes.

The S+ pass was performed as follows: The patient was placed in a supine position with the neck slightly extended. The patient underwent local anesthesia using 0.1-0.3 ml of 2% lidocaine, and the skin was disinfected with iodophor. The stylet was removed after the needle tip puncture was put into the nodule under ultrasound guidance. The needle was pulled out when the needle was cut back and forth 15-20 times within the nodule or when the sample material was seen in the hub. Suction was not used in the process. The material in the needle lumen was expelled onto one clean glass slide using a 5 ml air-filled syringe after each pass. All the steps were the same for the S+ and S- pass, except for the stylet removal step that was avoided in S- pass.

One slide specimen was made for each pass smear, and these slides were labeled as slides A1, B1, A2, and B2 in the order of pass. Sometimes slides A (A1 and A2) were two S+ specimens or two S- specimens since the choice of - the first pass was random to avoid bias of the pathologist. No on-site assessment was performed, where the specimens were air-dried, fixed in 95% alcohol, and stained with hematoxylin and eosin.

### Cytological evaluation

Five pathologists blinded to the stylet status of the passes evaluated cytology specimens. The same pathologist evaluated all specimens of one patient. Every thyroid FNA was first evaluated for specimen adequacy. Each specimen was classified as adequate or inadequate based on the Bethesda System for Reporting Thyroid Cytopathology (TBSRTC). Inadequate specimens (category I specimens in TBSRTC) were defined as specimens that did not meet the criteria for adequacy (presence of at least six groups of well-visualized follicular cells; each group containing at least 10 well-preserved epithelial cells).

The two S+ specimens and two S- specimens of one nodule were evaluated as a separate whole with a separate cytologic result (specimens A1 and A2 as “nodule A” specimens and specimens B1 and B2 as “nodule B” specimens). The S+ and S- specimens of the nodule were considered inadequate when the two S+ specimens and two S- specimens were inadequate.

### Outcome variables

Specimen adequacy was the primary outcome, while the yield for malignancy was the secondary outcome. Specimen adequacy was defined as the rate at which adequate specimens were obtained (proportion of non-TBSRTC category I specimens). The yield for malignancy was defined as a percentage of TBSRTC category VI specimens.

### Sample size estimation and statistical analysis

Power Analysis and Sample Size (PASS) version 11 was used for sample size estimation. The sample size was determined based on data from a meta-analysis involving 25,000 patients, which claimed an overall specimen adequacy rate of 87.1% for thyroid FNA (15). The specimen adequacy rate by S+ or S- FNA was then assumed to be 87.1%. The non-inferiority margin was then set at 10%, with a class I error of 0.025 (1 margin) and a class II error of 0.8. A total of 195 lesions per group were required assuming 10% dropouts. Besides, this study used its own control, and thus the final sample size was 195 nodules per group (195 nodules in total).

For a patient with more than one nodule undergoing FNA, the nodules were considered independent observations for statistical analysis. Continuous variables were expressed as means and standard deviations. Categorical variables were expressed as frequencies and percentages. The primary outcome was analyzed using Wald test. The difference between the adequacy of the two methods was expressed as a two-sided 95% confidence interval (CI). The S- method was considered non-inferior to the S+ method if the lower bound of the two-sided 95% (equivalent to one-sided 97.5%) CI of the difference in specimen adequacy (S- minus S+) was greater than -10%. Chi-square tests were used to analyze the secondary outcomes. Statistical Package for Social Sciences (SPSS) version 23 and Statistical Analysis System (SAS) version 9.4 were used for all analyses.

## Results

### Patients and nodules

**Figure 1:**
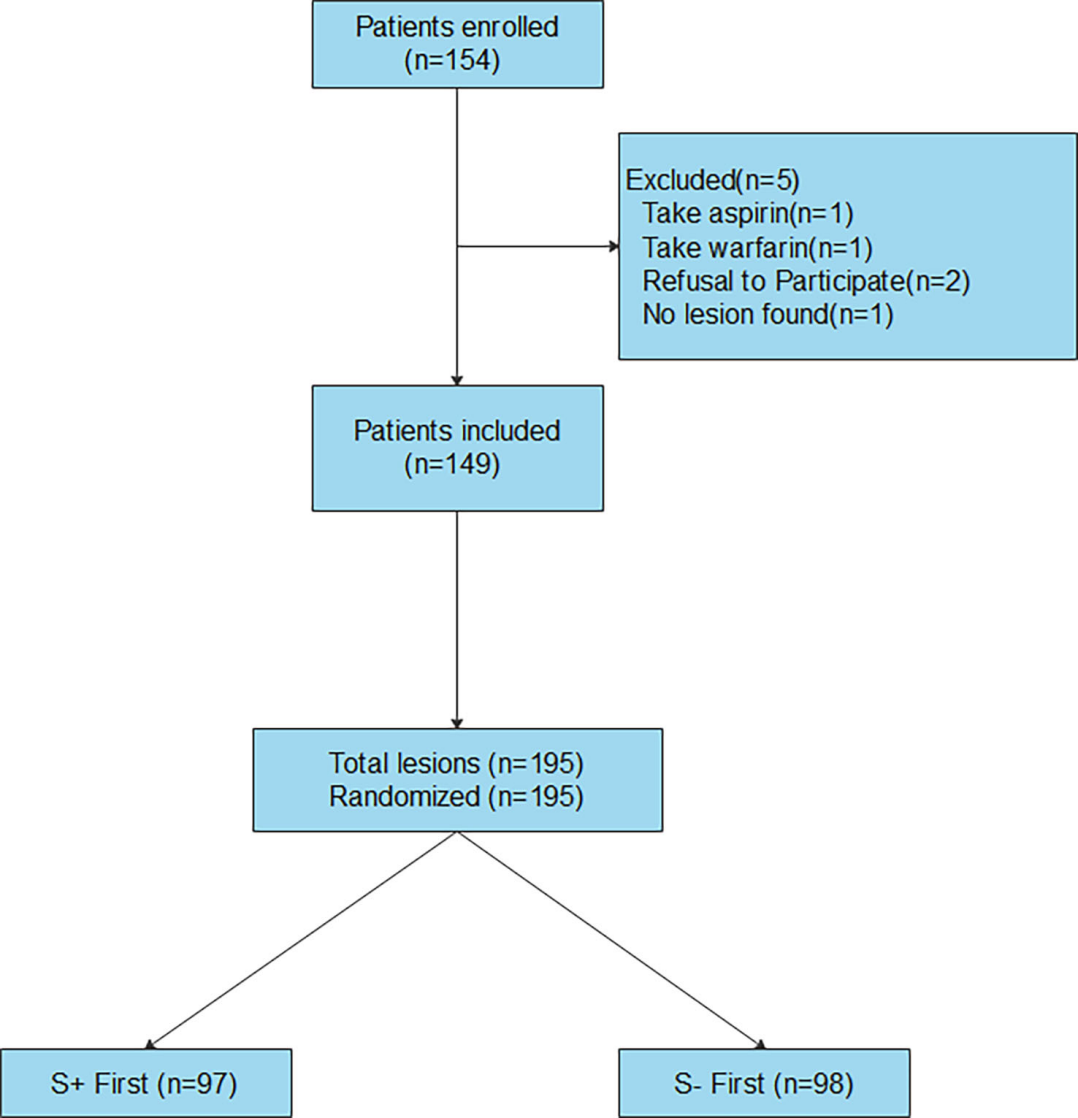
Flow diagram for 154 consecutive patients referred for thyroid nodules fine needle aspiration (FNA).

A total of 154 patients were enrolled from May 2022 to July 2022 (**Figure 1**). Five patients were excluded, of which 1 was taking aspirin, 1 was taking warfarin, 2 refused to participate, and nodules were not detected in one patient. Finally, 149 patients were included in the analysis (121 (81.21%) females and 28 males with 38 (25.5%) solitary nodules and 111 (74.5%) multiple nodules). The mean age of the included patients was 46.97 years (standard deviation (SD) 13.56). Only one nodule was sampled in 103 patients, and two nodules were sampled in 46 patients. A total of 195 nodules were sampled (mean nodule diameter, 1.42 cm; SD, 1.12 cm). The TIRADS category of the nodules was as follows: 3 in 52 (26.67%), 4a in 105 (53.85%), 4b in 33 (16.92%), and 4c in 5 (2.56%). The composition of the nodules was as follows: solid in 149 (76.41%) and solid-cystic in 46 (23.59%). The echogenicity of the nodules was hypoechoic in 161 (82.56%), isoechoic in 33 (16.92%), and hyperechoic in 1 (0.51%). A total of 101 nodules (51.79%) had calcifications. The vascularity of the nodules was peripheral in 26 (13.33%), central in 63 (32.31%), and no vascularity in 106 (54.36%). A total of 97 and 98 nodules were randomly selected for S+ first puncture and S- first puncture, respectively.

### Cytological results

The final cytological diagnosis was malignant (TBSRTC category VI) in 23 nodules (11.79%), suspicious for malignancy (TBSRTC category V) in 33 nodules (16.92%), follicular neoplasm or suspicious for a follicular neoplasm (TBSRTC category IV) in 5 nodules (2.56%), atypia of undetermined significance or follicular lesion of undetermined significance (TBSRTC category III) in 24 nodules (12.31%), benign (TBSRTC category II) in 91 nodules (46.67%), and inadequacy (TBSRTC category I) in 19 nodules (9.74%).

The two S+ specimens and two S- specimens of each nodule were evaluated as a separate whole, and the cytological results are shown in **Table 1**.

**Table 1:** Cytological results of specimens obtained with and without stylet.

TBSRTC category	S+(n=195)	S-(n=195)
I	28	26
II	89	90
III	23	23
IV	5	5
V	29	31
VI	21	20

### Specimen adequacy

The rates of obtaining adequate specimens were 167 of 195 nodes (85.64%) and 169 of 195 nodes (86.67%) for S+ and S- methods, respectively. The difference in specimen adequacy (S- minus S+) between the two methods was 1.03% (95% CI, -5.83% to 7.88%). The lower bound 95% CI of the difference in specimen adequacy (-5.83%) was greater than the predetermined non-inferiority margin of -10%, demonstrating the non-inferiority of the S- method to the S+ method.

Analysis of each subgroup showed that the differences in specimen adequacy were not significantly different between the two methods (**Table 2**).

**Table 2:** Subgroup analysis of specimen adequacy obtained with and without stylet.

Parameter	S+ adequacy no. (%)	S- adequacy no. (%)	*P* value
Size			
<1cm(n=96)	80(83.33%)	81(84.38%)	0.845
1-3cm(n=82)	70(85.37%)	71(86.59%)	0.822
>3cm(n=17)	17(100%)	17(100%)	1.000
Depth in thyroid			
First third(n=132)	115(87.12%)	116(87.88%)	0.852
Middle third(n=52)	42(80.77%)	43(82.69%)	0.800
Last third(n=11)	10(90.91%)	10(90.91%)	1.000
Echogenicity			
Hypoechoic(n=161)	139(86.34%)	142(88.20%)	0.616
Isoechoic(n=33)	27(81.82%)	26(78.79%)	0.757
Hyperechoic(n=1)	1(100%)	1(100%)	1.000
Calcifications			
Microcalcification(n=60)	52(86.67%)	53(88.33%)	0.783
Macrocalcification(n=41)	31(75.61%)	34(82.93%)	0.414
No calcification(n=94)	84(89.36%)	82(87.23%)	0.650
Vascularity			
Central(n=63)	56(88.89%)	55(87.30%)	0.783
Peripheral(n=26)	20(76.92%)	23(88.46%)	0.465
None(n=106)	91(85.85%)	91(85.85%)	1.000
Composition			
Solid(n=149)	128(85.91%)	131(87.92%)	0.606
Solid-cystic(n=46)	39(84.78%)	38(82.61%)	0.778
TIRADS category			
3(n=52)	44(84.62%)	43(82.69%)	0.791
4a(n=105)	92(87.62%)	92(87.62%)	1.000
4b(n=33)	27(81.82%)	30(90.91%)	0.475
4c(n=5)	5(100%)	5(100%)	1.000

### Yield for malignancy

The yields for malignancy were 21 of 195 nodules (10.77%) and 20 of 195 nodules (10.26%) for S+ method and S- method, respectively (*P* = 0.869). Similarly, the yields for malignancy were also not significantly different between the two methods when suspicious malignant nodules were included in the malignancy group (S+:50/195(25.64%) vs. S-:51/195(26.15%), *P* = 0.908).

### Adverse events

No complications or needle blockage were reported in this study.

## Discussion

Puncture needles with a stylet is widely used by many puncturers during thyroid FNA (1, 2) based on the unproven premise that using the stylet improves the specimen’s quality by preventing the needle’s lumen from being blocked or contaminated by other non-lesion components, including blood, before entering the target lesion. Nevertheless, stylet is time-consuming and labor-intensive and may also increase the risk of accidental needle stick injuries. Meanwhile, a needle with a stylet is expensive compared to a needle without a stylet, such as an ordinary syringe needle, and thus may significantly increase the cost of puncture. However, recent data have shown that using a stylet in other areas of FNA, such as gastrointestinal endoscopic FNA and respiratory endoscopic FNA, does not improve the quality of specimen (3–8). Besides, stylet is sometimes associated with an inferior specimen quality (9).

Besides, there are limited data comparing thyroid FNA with and without a stylet. A published trial comparing the two techniques compared only a subset of selected thyroid nodules (hypoechoic vascular type II nodules) (1), representing only a small fraction of the total types of thyroid nodules (14). No study has evaluated the value of stylet for the puncture of all types of thyroid nodules. To the best of our knowledge, this is the first study assessing the value of the stylet in overall unselective thyroid nodules. In this study, results showed that a stylet does not improve specimen adequacy or yield for malignancy during thyroid FNA, consistent with results in other areas of FNA.

However, a previous published study comparing thyroid FNA with and without a stylet showed that a stylet can improve specimen adequacy (1), by preventing blood or cystic fluid from entering the lumen of the needle during needle insertion into the target nodule. In this study, results showed that stylet may prevent puncture blood from entering the lumen of the needle before removal of the stylet, but this may not enhance specimen quality conducted by experienced puncture operators. Experienced operators can often anticipate the puncture route and angle, and the needle can be punctured into the target nodule with no or only slight adjustment. Nonetheless, puncture bleeding from thyroid tissue often occurs during repeated needle punctures during a long adjustment period. Meanwhile, the process of removal of the stylet generates a negative pressure (11), which depends on the speed and the length of removal. Animal experiments have shown that the maximum negative pressure can reach close to one atmosphere (10), which may affect specimen quality. The needle tip cut has not yet been used to obtain lesion cells when the stylet is withdrawn after the puncture needle has entered the target nodule. The negative pressure generated by the withdrawal of the stylet may draw non-lesion components, such as blood and cystic fluid, into the lumen of the needle, which can interfere with the entry of lesion cells obtained by subsequent cutting. Previous gastrointestinal endoscopic FNA studies also highlighted the above when there were significantly higher bloody specimens and lower adequate specimens in the S+ group than in the S- group (9).

Furthermore, only 480 of 2750 patients were screened in the study comparing thyroid FNA with and without a stylet (1) rather than including patients consecutively. Besides, different needles were used in different nodules, and the study lacked true randomization. The above may have contributed to selection bias, possibly by artificially assigning easier satisfactory nodules to the experimental group or excluding more difficult nodules from the experimental group.

There is no doubt that the higher cost of puncture needles with a stylet than those without a stylet, such as an ordinary syringe needle, will significantly increase the puncture cost for patients. Therefore, its higher cost of puncture needles with stylet is unjustified if it does not improve specimen quality. Considering a large number of FNA examinations performed worldwide each year, the cost issue is not irrelevant. Our findings provide a reasonable basis for using low-cost puncture needles without a stylet can be used during thyroid FNA, which would reduce the average per-patient puncture cost and save health insurance funds.

In this study, the patient population was representative of the typical patient population seen by most thyroid surgeons working in tertiary referral centers, with most being women and a wide age range. Therefore, the results may broadly apply to most patients with thyroid nodules. FNA is an operation-dependent procedure. Herein, only one experienced operator performed the procedures, thus excluding the influence of inter-operator differences. Furthermore, a non-inferiority design was used to ensure an adequate sample size. A randomized design was used for the order of S+ first or S- first puncture of each nodule to exclude the effect of bleeding from the first puncture on the adequacy of the second specimen. Patients using anticoagulant therapy, such as aspirin and warfarin, were excluded since these treatments affect specimen adequacy (16). All possible interference factors were eliminated to ensure that other factors, except the stylet, do not affect the results.

This study has some limitations. First, the operator was not blinded to the stylet status of each puncture since this is logically impossible. Second, five cytopathologists interpreted the slides, and interobserver variation may have occurred during the interpretation. However, all slides from one patient were evaluated by the same cytopathologist. Third, only 25-gauge needles were used, and thus these results may not apply to other needle sizes. Finally, the sensitivity, specificity, and accuracy of the two techniques were not explored.

## Conclusions

In conclusion, this non-inferiority study demonstrated that the absence of a stylet during thyroid FNA is non-inferiority to using a stylet on specimen sampling. If other studies confirm these results, using a low-cost needle without a stylet during thyroid FNA is justified. This would make the whole process easier, as well as more cost-effective.

## Data availability statement

The raw data supporting the conclusions of this article will be made available by the authors, without undue reservation.

## Ethics statement

This study was approved by the Ethics Committee of Fuyang People’s Hospital (NO: 2022-80). The patients/participants provided their written informed consent to participate in this study.

## Author contributions

All authors contributed to the study conception and design. PL, XM, and WM performed material preparation, data collection and analysis. PL wrote the first draft of the manuscript and all authors approved previous versions of the manuscript. All authors contributed to the article and approved the submitted version.
